# Individual Navigation Templates for Subcortical Screw Placement in Lumbar Spine

**DOI:** 10.17691/stm2021.13.5.05

**Published:** 2021-10-29

**Authors:** R.A. Kovalenko, V.A. Kashin, V.Yu. Cherebillo

**Affiliations:** Assistant, Department of Neurosurgery Pavlov First Saint Petersburg State Medical University, 6-8 L’va Tolstogo St., Saint Petersburg, 197022, Russia; PhD Student, Department of Neurosurgery Pavlov First Saint Petersburg State Medical University, 6-8 L’va Tolstogo St., Saint Petersburg, 197022, Russia; Professor, Head of the Department of Neurosurgery Pavlov First Saint Petersburg State Medical University, 6-8 L’va Tolstogo St., Saint Petersburg, 197022, Russia

**Keywords:** navigation template, navigation guide, 3D printing, subcortical trajectory

## Abstract

Subcortical screw placement is currently performed using frontal view fluoroscopy or intraoperative O-arm navigation system. The emergence of a novel technique for spinal navigation based on individual navigation templates created using 3D printing technology determines the need to study their safety and effectiveness in subcortical implantation.

**The aim of the study** was to evaluate and compare the efficacy of subcortical implantation of pedicle screws in the lumbar spine using individual navigation templates versus intraoperative fluoroscopy.

**Materials and Methods:**

The study was based on the analysis of treatment results in 39 patients who underwent surgery with subcortical implantation of 130 screws using the MidLIF technique. In group 1, navigation templates were used, in group 2 — intraoperative fluoroscopic control. Comparative analysis of implantation correctness and time, the total operation time, and radiation load was performed.

**Results:**

The mean distance between the screw and the cortical plate recorded in the groups ranged within 1.20–3.97 mm, without statistically significant difference (p>0.05). The mean time of pedicle screw implantation was 137.0 [115.25; 161.50] s in group 1 and 314.0 [183.50; 403.25] s in group 2. The total operation time was reduced from 173.0 [155.0; 192.25] min in group 2 to 119.0 [108.0; 128.75] min in group 1. The average of 1.0 [1.0; 2.0] X-ray image was performed to place one screw in group 1, while it was 12.0 [10.0; 13.25] in group 2. The differences between the groups in terms of implantation time and radiation load were statistically significant (p<0.05).

**Conclusion:**

Compared with intraoperative fluoroscopy, the use of individual navigation templates for subcortical implantation of pedicle screws provides their correct positioning with a significant reduction in both operation time and radiation load at similar safety.

## Introduction

The main advantages of the subcortical trajectory for placement of pedicle screws, as compared to the classical one, are stronger fixation and a more medial location of the insertion point, which makes it possible to reduce the dissection area and make the access smaller and less traumatic versus the classical midline approach. It is believed that stronger fixation is achieved due to the proximity of the screw to the cortical bone throughout its entire length [1–7]. Subcortical screw placement is currently performed using frontal view fluoroscopy or intraoperative O-arm navigation system.

One of the directions of using 3D printing in spinal surgery is application of individual navigation templates (INT), which allow pedicle screw implantation with high accuracy and safety. Some authors [8–10] have analyzed the possibilities of using this innovative technology for screw implantation at the subcortical trajectory, but we failed to find any comparative analyses of the results of applying this technique versus intraoperative fluoroscopy in a randomized study.

**The aim of the study** was to evaluate the efficacy of subcortical implantation of pedicle screws in the lumbar spine using 3D printed individual navigation templates versus intraoperative fluoroscopy.

## Materials and Methods

The study involved analyzing the results of implantation of 130 pedicle screws placed in 39 patients aged 37 to 71 years during decompression and stabilization surgery in the lumbosacral spine using MidLIF technology. The study complies with the Declaration of Helsinki (2013) and was performed following approval by the Ethics Committee of Pavlov First Saint Petersburg State Medical University (Russia). Written informed consent was obtained from each patient.

**Figure 1.**
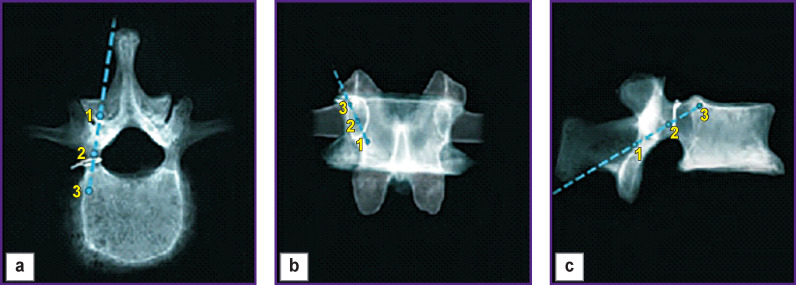
Trajectory of the pedicle screw insertion in the lumbar spine along the subcortical trajectory: (a) axial projection; (b) frontal projection; (c) sagittal projection; *1* — insertion point, *2* — center of pedicle, *3* — position of the screw tip

The screws were placed on the subcortical trajectory: from the inferomedial to the superolateral direction of the pedicle (Figure 1).

Two groups of patients were formed by randomization method using random numbers: group 1 — implantation using INT (n=19), 66 screws; group 2 — implantation with intraoperative fluoroscopic control (n=20), 64 screws.

A channel in the vertebral pedicle was formed using a drill 2 mm in diameter or a high-speed drill with a 1–2 mm bur. A polyaxial screw 4.35 mm in diameter and 35–40 mm in length was implanted. When using INT, the trajectory was planned according to the principle of the maximum proximity of the screw to the cortical bone of the pedicle, placing the screw tip under the endplate in its lateral part.

**Figure 2.**
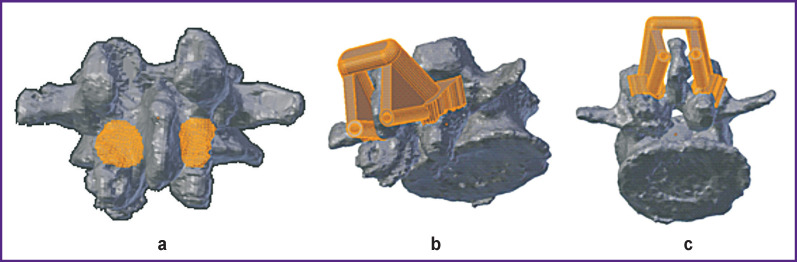
Location of the support zone (a) and design of navigation templates (b), (c) for transpedicular fixation along the subcortical trajectory

In group 1, monolateral single-level INT were used in 5 cases, while bilateral single-level INT were used in 14 cases. A part of the vertebral arch was used as a support platform (Figure 2). The inner diameter of the tube was 3 mm, the outer diameter was 5 mm. Two basic elements including a support platform and a guide tube were connected by a transverse beam.

**Figure 3.**
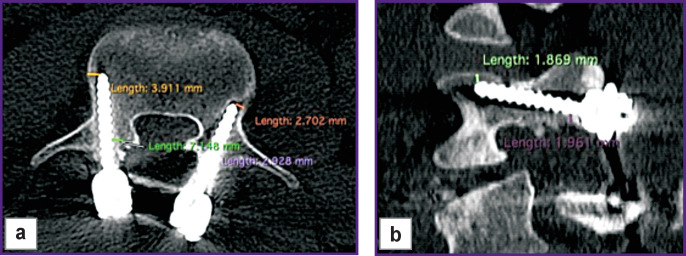
Assessment of the correct position of the pedicle screw along the subcortical trajectory: (a) distance between the cortical bone and the screw in the axial plane; (b) distance in the sagittal plane

To assess the correctness of the screw position, there was developed a system of analysis based on the following criteria:

the minimum distance to the medial border of the pedicle at the insertion point and the distance from the lateral edge of the vertebral body to the screw tip were calculated for each screw in the axial plane;

the distance from the screw to the lower edge of the pedicle at the insertion point and the distance from the upper edge of the vertebral body to the screw tip were calculated in the sagittal plane (Figure 3).

Evaluation criteria in the compared groups included implantation safety based on the disruption of cortical layer continuity, the frequency of complications, the number of revision interventions, the distance from the screw to the cortical layer, deviation degree when using INT, the number of X-rays taken and the time spent on both implantation and the entire operation.

The time of implantation was recorded from the moment of completing the operative access to complete placement of all screws. In cases when after application of the template it was necessary to perform additional skeletonization of the surface due to unsatisfactory contact, this period was also counted as time spent on implantation.

### Statistical processing

The data obtained were processed using the Statistica 10.0 software. All samples used were represented by non-normal distributions, which was confirmed by graphical methods as well as using the Kolmogorov–Smirnov and Shapiro–Wilk tests. In this regard, the data were presented as a median, 1^st^ and 3^rd^ quartiles. Differences between the groups were assessed using the Mann–Whitney U-test and the Kruskal–Wallis test for two independent samples; the differences were considered statistically significant at p<0.05.

## Results

According to implantation safety analysis, in group 1 (INT), there were four cases of cortical bone perforation, one case of cranial pedicle perforation, two cases of perforation of the lateral wall of the vertebral body by the screw tip, and one case of endplate perforation by the screw tip. According to CT data, one patient underwent revision with reimplantation (replacement) of a screw (endplate perforation with a significant exit of the screw into the cavity of the overlying intervertebral disc). During caudal migration at the pedicle level, the patient had no radicular symptoms after surgery, therefore the screw was not replaced.

In group 2 (fluoroscopy), 5 cases of perforation were revealed. Three perforations of the cortical layer were noticed at the level of the pedicle: cranial, caudal, and lateral. In two cases, the screw tip exceeded the bone at the level of the vertebral body: disruption of the endplate continuity and lateral exit of the screw. The differences between the groups in the number of perforations were not statistically significant. Repeated operations in order to replace the screws were not performed in group 2. Differences in the number of reoperations due to incorrect implantation were not statistically significant, p>0.05.

No damage to neural structures was recorded in any of the groups.

Assessment of the distance between the screw and the cortical bone at the control points is presented in the Table. The deviation of the insertion point in the axial plane in the lateral direction is largely determined by the lacking possibility to insert the screw at the maximum medial point and maintain the necessary axial implantation vector without resection of the spinous process.

**Table:** Distance between the implanted screw and the cortical bone (mm), Me [Q1; Q3]

Control points	Group 1 (66 screws)	Group 2 (64 screws)
Point 1 — the axial plane, the entry of the pedicle	4.64	4.22
	[3.32; 5.97]	[2.57; 6.50]
Point 2 — the axial plane, the screw tip	0.92	3.89
	[0.55; 1.23]	[2.87; 6.03]
Point 3 — the sagittal plane, the entry of the pedicle	2.43	6.26
	[1.23; 3.21]	[4.67; 7.87]
Point 4 — the sagittal plane, the screw tip	1.04	0.94
	[0.45; 1.95]	[0.45; 1.42]

Statistically significant differences in the distance with a lower value were obtained when using INT in the axial plane in the area of the screw tip (see the Table, point 2). A similar tendency was noted at the third control point: in the sagittal plane, the screw was located farther from the lower edge of the pedicle in group 2. At the endpoint in the sagittal plane, there were no significant differences between the groups; in most cases, the screw tip was located in the immediate proximity to the endplate.

Deviation analysis at all control points showed the average deviation from the planned trajectory within 0.9–6.3 mm, which can be regarded as an acceptable indicator taking into account the morphometric parameters of lumbar spine pedicles. At the insertion point, the deviation was mostly registered in the sagittal plane, at the end point — in the axial plane; the difference between the groups was not statistically significant (p>0.05).

The average time of pedicle screw placement was 137.0 [115.25; 161.50] s in group 1 and 314.0 [183.50; 403.25] s in group 2. The average operation time was 119.0 [108.0; 128.75] min in group 1 and 173.0 [155.0; 192.25] min in group 2 (p<0.05).

When installing one screw, the average of 1.0 [1.0; 2.0] X-ray images were performed in group 1 and 12.0 [10.0; 13.25] images in group 2 (p<0.05).

## Discussion

Subcortical implantation of pedicle screws in the lumbar spine is becoming more and more popular due to its minimal invasiveness and greater strength compared to traditional transpedicular fixation [1, 11, 12].

At present, the most common technique is screw placement using intraoperative fluoroscopy in frontal and lateral projections or CT navigation [13]. However, these techniques expose both the patient and the medical staff to additional radiation. Besides, the use of intraoperative CT navigation is limited by the high cost of the required equipment.

The actively developing technique of INT manufactured using 3D printing technologies has become the subject of multiple studies devoted to its application in all parts of the spine [14–21]. The use of templates in the lumbar spine is limited by the anatomical features of this area, namely, the paravertebral muscles preventing the adequate positioning of the INT when choosing the classical transpedicular trajectory of implantation [22, 23].

In this regard, the vector of INT application for the lumbar spine was reoriented to the use of non-classical trajectories. For example, Shao et al. [23] have proposed an INT version for placement of transpedicular transdiscal screws when fixing one spinal motion segment. Cao et al. [24] have proposed an INT design for contralateral translaminar-transarticular fixation.

Along with these studies, there is a promising direction of research devoted to the use of a subcortical trajectory, in which the pressure of the paravertebral muscles is minimized and, as a consequence, the correctness of INT application increases.

Kaito et al. [8] have conducted a cadaver study, which showed the high efficiency of INT use for subcortical implantation. 91.4% of the screws were completely in the bone structures, though cortical bone perforation was less than 2 mm in all cases.

In 2018, Kim et al. [9] presented a clinical case of subcortical screw placement, demonstrated the convenience of this technique and the importance of preoperative planning, underlining the importance of further study of the technique effectiveness and accuracy in larger samples.

Marengo et al. [10] demonstrated a clinical study of single-segment fixation results in 11 patients (44 screws). The average deviation of the trajectory was 0.91 mm, while the deviation of degree A (<2 mm) was noted only in two cases.

According to our study, the use of INT for screw implantation in the lumbar spine was not accompanied by an increase in implantation safety and did not affect the number of reoperations associated with incorrect screw placement as compared to the use of intraoperative fluoroscopy. Screw placement with the aid of navigation templates contributes to the achievement of a more correct trajectory and a closer position of the screw to the cortical bone, which, hypothetically, should have a favorable effect on the stability of the structure and reduce the risk of subsequent bone resorption around the screw. Our experience has shown that when using fluoroscopic control (even in the case of biplanar fluoroscopy) it is extremely difficult to form an implantation trajectory ensuring the screw tip to be located in close proximity to the cortical bone in two planes. Consequently, implantation correctness should be higher than with intraoperative CT navigation, which allows assessing the insertion trajectory in three planes before the start of insertion.

**Figure 4.**
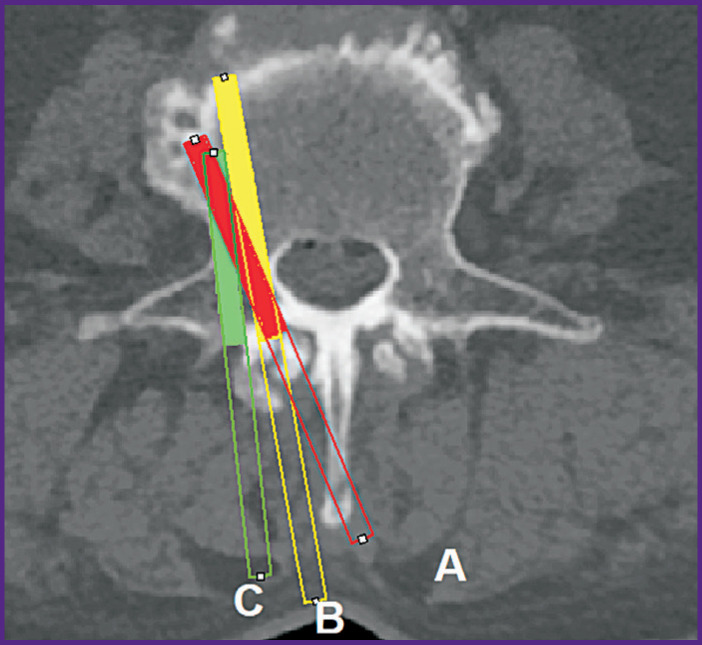
Various options for planning the subcortical trajectory in the axial plane in the lumbar spine

When planning the trajectory, the surgeon has to choose between three different options:

a more medial location of the insertion point with the most correct implantation axes in conjunction with the removing a part of the spinous process (Figure 4, option *A*);

a more medial location of the insertion point without resection of the spinous process, combined with a decrease in the axial angle of implantation, i.e. more “straight” insertion of the screw with a greater distance from the cortical bone at the end point (Figure 4, option *B*);

lateral displacement of the insertion point in observance of implantation correctness at other control points (Figure 4, option *C*).

Studying the possibilities of using INT, we tested all three implantation variants, of which the latter was preferred since the resection of the spinous process could serve as an unfavorable biomechanical factor due to the removal of the ligamentous apparatus, while a more straight trajectory is associated with a greater distance from the cortical bone in the end point.

The use of INT for transpedicular fixation along the subcortical trajectory in the lumbar spine is also accompanied by a statistically significant decrease in the time of implantation and radiation exposure and promotes achievement of a more correct trajectory of the screw.

## Conclusion

Compared with intraoperative fluoroscopy, the use of individual navigation templates for subcortical implantation of pedicle screws provides their correct positioning with a significant reduction in both operation time and radiation load at similar safety.
